# Letter to the Editor: “Development and application of an intelligent platform for automated recognition of surgical instruments in laparoscopic procedures: a multicenter retrospective study”

**DOI:** 10.1097/JS9.0000000000002726

**Published:** 2025-06-18

**Authors:** Ping Zhao, Hao Sun, Qiuwan Han

**Affiliations:** a Department of Anesthesiology, Hangzhou Xiaoshan District Orthopedics Hospital of Traditional Chinese Medicine, Hangzhou, Zhejiang, China; b Wolfson Institute for Biomedical Research, UCL Division of Medicine, University College London., London, UK

*Dear Editor,*

We read with great interest the recent article published in the *International Journal of Surgery*. Hu *et al*^[1]^. The authors proposed a novel surgical instrument recognition model that integrates transformer and CNN architectures, and explored its potential applications in various clinical scenarios such as intraoperative assessment and postoperative teaching. The study demonstrates a degree of innovation and practical relevance. However, after a careful review, we believe that several aspects related to methodological design, model evaluation, and validation of clinical utility warrant further refinement. We respectfully offer the following comments:

First, regarding dataset construction, while the authors developed a relatively large dataset, basic demographic information such as patient age, sex, and disease type was not provided. This omission raises concerns about potential bias during model training and may affect the generalizability of the system across different populations and institutions. Additionally, the manuscript lacks clarity on how the model performs across different hospitals or surgeons, and there is no systematic evaluation of its generalization capacity.

Second, in terms of clinical application, the authors deployed the model via the SurgSmart platform and developed multiple functions, including quick review mode, instrument reports, heatmap visualization, and a teaching module. Although user feedback through preliminary questionnaires appears positive, the evaluation remains largely subjective. Objective clinical outcomes such as reductions in operative time, changes in intraoperative complication rates, or improvements in teaching assessment scores were not reported, limiting the assessment of practical impact. Furthermore, the study does not explore how instrument recognition might be integrated with key intraoperative events, such as bleeding or suture failure, to enable real-time alerts or decision support, which is a crucial value proposition of AI-assisted surgical systems.

Third, regarding the interpretation of instrument recognition results, the authors suggest that the detection of certain tools (e.g., clips) may aid in localizing critical surgical steps like clipping the cystic duct or artery. However, we believe this observation more likely reflects the natural association between specific instruments and specific tasks—the instrument use is a consequence rather than a cause of the surgical step. Using tool detection as a proxy for surgical phase identification may therefore risk reversing the causal inference, which requires further scrutiny and justification.

Lastly, in model evaluation, the authors primarily used frame-level metrics to assess recognition performance but did not sufficiently address temporal stability. Given the sequential nature of surgical videos, a reliable instrument recognition model should demonstrate robust temporal consistency to avoid issues like frame-skipping or misclassification. We recommend incorporating video-level metrics such as recognition stability and temporal smoothness in future evaluations to provide a more comprehensive assessment.

In summary, this study presents an insightful exploration into surgical instrument recognition and its potential clinical applications. Nonetheless, enhancements in model generalizability, evaluation metrics, and clinical validation are needed to further support real-world implementation. We encourage future work to strengthen cross-scenario adaptability, build task-specific quantitative assessment frameworks, and conduct comparative analysis with existing models^[2]^ (e.g., EndoNet^[3]^, SV-RCNet^[4]^) to advance the clinical translation of such technologies. Finally, we commend the authors for leveraging surgical videos for secondary analysis, which will serve as a valuable reference for future research in this area.

## Data Availability

Not applicable.
